# Designing a Community Engagement Framework for a New Dengue Control Method: A Case Study from Central Vietnam

**DOI:** 10.1371/journal.pntd.0002794

**Published:** 2014-05-22

**Authors:** Darlene McNaughton, Thi Thu Huong Duong

**Affiliations:** 1 Discipline of Public Health, Flinders University, Adelaide, Australia; 2 Department of Sociology, University of Journalism and Communication, Hanoi, Vietnam; Duke-National University of Singapore, Singapore

## Abstract

**Background:**

The *Wolbachia* strategy aims to manipulate mosquito populations to make them incapable of transmitting dengue viruses between people. To test its efficacy, this strategy requires field trials. Public consultation and engagement are recognized as critical to the future success of these programs, but questions remain regarding how to proceed. This paper reports on a case study where social research was used to design a community engagement framework for a new dengue control method, at a potential release site in central Vietnam.

**Methodology/Principal Findings:**

The approach described here, draws on an anthropological methodology and uses both qualitative and quantitative methods to design an engagement framework tailored to the concerns, expectations, and socio-political setting of a potential trial release site for *Wolbachia*-infected *Aedes aegypti* mosquitoes. The process, research activities, key findings and how these were responded to are described. Safety of the method to humans and the environment was the most common and significant concern, followed by efficacy and impact on local lives. Residents expected to be fully informed and engaged about the science, the project, its safety, the release and who would be responsible should something go wrong. They desired a level of engagement that included regular updates and authorization from government and at least one member of every household at the release site.

**Conclusions/Significance:**

Results demonstrate that social research can provide important and reliable insights into public concerns and expectations at a potential release site, as well as guidance on how these might be addressed. Findings support the argument that using research to develop more targeted, engagement frameworks can lead to more sensitive, thorough, culturally comprehensible and therefore ethical consultation processes. This approach has now been used successfully to seek public input and eventually support for releases *Wolbachia*-infected mosquitoes, in two different international settings - Australia and Vietnam.

## Author Summary

In recent years, a number of new strategies using novel technologies for the control of dengue fever control have emerged. These strategies are notably different from their predecessors and not without controversy. Many also require open release field trials to test their efficacy. Public consultation and engagement are recognized as critical to the future success of these programs, but questions remain regarding how to proceed. In this paper we describe an approach to public engagement that uses social research to design an engagement framework and communication materials tailored to the concerns, expectations, and socio-political setting of potential trial release sites. This approach was developed and implemented in Australia (2008–2010) where the first publicly supported field trials occurred January 2011. We report here on the implementation of this approach in Vietnam (2009–2010) where the second release will occur in 2014. This paper describes the process, research activities, outcomes and key findings from the Vietnamese field site. It highlights key public concerns and expectations about engagement and authorization and shows how these were used to develop a more targeted, culturally appropriate and comprehensible engagement framework and communication materials. The paper demonstrates the viability of this approach to community engagement for new dengue control strategies, in a ‘developing’ country context.

## Introduction

The *Wolbachia* strategy aims to ‘manipulate mosquito populations to make them incapable of transmitting dengue viruses between people’ (www.eliminatedengue.com). Its potential emerged following the successful transference of the insect bacterium *Wolbachia pipientis* from the fruit fly *Drosophila melanogaster* into the *Aedes aegypti* mosquito [1], [2], [3]. Later studies showed that the bacterium spread effectively into wild populations, had a life-shortening effect on the mosquito, blocked the development of some dengue viruses and some strains had a life-shortening effect on the mosquito [4], [5]. These properties would, in all likelihood, greatly reduce the mosquito's capacity to transmit the virus. To trial its effectiveness in real world conditions, required a series of field release through which *Wolbachia*-infected mosquitoes would be released into wild populations the aim being to replace these.

The *Wolbachia* method is one of several strategies to emerge in recent year that use a range of new technologies to combat dengue fever. While some focus on genetic modification, others, like *Wolbachia*, use biological control [1], [5], [6]. However, these strategies are very different from their predecessors, notably source reduction and insecticide use, and are not without controversy. Moreover, many require open field releases to test their efficacy and potential uses. Significantly, these need to occur in the locations where dengue vectors are found, most commonly the homes, and places of work, education, worship and leisure of local residents at a release site.

Most commentators recognize that the political and ethical complexities of community field trials are considerable and that public and government approval in conjunction with high quality science are of central importance. It is also widely acknowledged, that given the spread and increasing prevalence of dengue fever throughout the tropics, field trials will need to be undertaken in a variety of locales, regions and countries, both so called developed and developing. While public engagement is also recognized as critical to the use and future success of these strategies, many questions remain regarding how to proceed in ways that are ethical, and comprehensible to those being asked to trial these strategies in their homes and backyards.

In 2008 an approach to engagement drawing on anthropological methodologies and insights was developed for the *Wolbachia* strategy. It was implemented in Cairns, Australia from 2008–2010 [6] and in January 2011 the first field release of *Wolbachia-*infected *Ae. aegypti* commenced. Drawing on anthropological methodologies and insights, this approach recognizes that different communities will have divergent expectations, knowledge, concerns, political structures and cultural sensibilities, that need to be understood *and* taken into account, if one is to engage sensitively, ethically and effectively [6], [7], [8], [9], [10], [11] [12]. The most reliable way to do this, is to talk with residents at a potential release site about the new dengue control methods and ask what their concerns are, how they want to be engaged and what would constitute authorization [6], [11]. From this research, an engagement framework is developed that is sensitive to local needs, expectations, knowledge and concerns.

So, rather than simply adopting an engagement strategy that was developed elsewhere and implementing it in another setting, this approach uses social research to design an engagement framework and communication materials that are tailored specifically to potential release sites. In brief, it begins by undertaking systematic social research to: (a) document the socio-political context and identify the various publics and stakeholders at the potential release site, (b) determine how they want or expect to be engaged and the forms this should take, (c) explore what would constitute authorization, (d) identify any questions or concerns they might have about the *Wolbachia* strategy, (e) identify lay knowledge of the disease, its transmission, vectors, perceived risk, etc. and (f) develop responses to these. The results of this research are then used to *design* a community engagement framework tailored specifically to the sociopolitical setting, and the requirements and expectations of a given population [6].

This paper describes the use of this approach from June 2009 to September 2010 at the *second* potential *Wolbachia* release site - Tri Nguyen Island, in central Vietnam. It outlines the process, research activities, outcomes and key findings from the Vietnamese field site. It also highlights key public concerns and expectations about engagement and authorization and shows how these were used to develop a more targeted, culturally appropriate and comprehensible engagement framework and communication materials. Most significantly, the paper demonstrates the viability of this approach to community engagement for new dengue control strategies, in a ‘developing’ country context. It is hoped that by reporting on the methodology, process and results, that readers will be able to see the steps taken and assess the capacity of this approach to reflect and address local requirements and expectations, as well as its potential applicability to other programs.

## Methods

### The setting

Dengue fever has a long history in Vietnam and continues to represent a major public health problem [13]. Disease transmission occurs throughout the year in the south of the country but is limited to the warmer months in the northern and highland areas. Two vectors are active in disease transmission, the *Ae. albopictus* and *Ae. aegypti* mosquitoes [12] [14], [15]. Historically, dengue control in Vietnam has focused on source reduction, container management, insecticides and community mobilization – the later relying on household visits by collaborators and the management of water storage containers [15]. Since 1989, community-based biological control initiatives using *Mesocyclops spp.* to control mosquito breeding in household water containers have also been introduced [15], [16], [17], [18]. These have also included successful community mobilization around the management of water storage containers and the presence of *Mesocyclops spp*.

**Figure 1:**
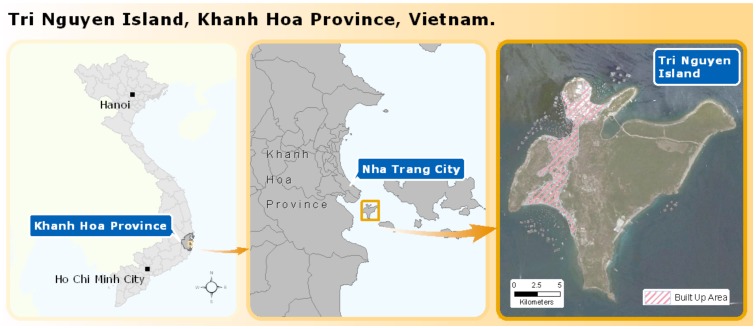
Map of study region. Tri Nguyen Island, Khanh Hoa Province, Central Vietnam.

Tri Nguyen Island (TNI) or Hon Mieu (‘Island Shrine’), as it was known historically, is located to the southeast of the city of Nha Trang (NT) in Khanh Hoa province, central Vietnam (Figure 1). It was selected as a *potential* release site for the *Wolbachia* strategy for a number of reasons. These include its physical isolation, its proximity to the Pasteur Institute in Nha Trang, famous for its work on infectious diseases, and residents' previous involvement in mosquito ecology and vector studies. Since the late 1940s and during the war with France, people from other provinces such as Quang Nam, Quang Ngai, Binh Dinh, Phu Yen moved to TNI. Today the island is stratified into 3 hamlets each with its own leader, which together represents one sector of the Vinh Nguyen ward of Nha Trang city, in Khanh Hoa province. In 2009 the population of TNI was 3253 residents, living in 710 households spread across three hamlets, each of which had its own political leaders [19].

### Methods

The social research activities described here were undertaken over 16 months (June 2009—September 2010) and included six weeklong fieldtrips to Tri Nguyen Island. Research activities centered on two key groups: a) Residents of Tri Nguyen island and b) health providers, government officials and scientists with responsibilities at the local, regional and national levels (hereafter, Leaders). It is widely established that qualitative research methods are the most appropriate for assessing the views of a population, in part because of their emphasis on context and their documentation of knowledge and attitudes in a given geopolitical setting. In this study, key or recurring themes from the qualitative research were explored further using quantitative measures (a household survey and anonymous questionnaire) and results were triangulated (compared, challenged or confirmed) across different methods: interviews, observations, questionnaires and a series of community meetings and workshops - styled on a focus group. Importantly, the findings presented here should not be seen as isolated research activities, but as a body of interconnected data developed over time using iterative processes and then contextualized, triangulated and crosschecked. An overview of the research activities undertaken at each phase, the issues they explored, how participants were recruited and the outputs they produced, is provided in Table 1.

**Table 1:** Process, issues, methods and recruitment.

Steps in the Process	Methods used	Target Population	Recruitment	Analysis
***Phase 1. Staff training***	Training Vietnamese staff: dengue, *Wolbachia* entomology, social research	Entomologists, Anthropologist	None	
*Outcomes*	Social science staff (2) develop a literature and database on peer reviewed & grey literature on dengue history, management, bio-control, GM in Vietnam and internationally.			
***Phase 2. Socio-political context: Governmental and of release Baseline data – qualitative***	1^st^ Workshop with Leaders. Sought advice/input on nature and from of engagement & authorization, socio-political structures, key stakeholders, communication materials, initial responses to *Wolbachia* strategy	Senior national, provincial, district and commune leaders, scientists and health providers	Purposive: Identified by senior Vietnamese Project staff, representatives from MoH.	Responses recorded. Anon. Questionnaire distributed and analyzed
	Introduction of social science staff to TNI community and leaders, September 2009	Residents and Leaders of TNI	Leaders known to senior entomologist.	Recorded as field notes.
	In-depth interviews (A) (n = 10) Aims: history, socio-political structure, engagement, demographics, dengue history of TNI, September 2009	Local leaders TNI & residents with specific knowledge of these issues.	Purposive: Identified by senior entomologist	Audio recorded Analysis in NVivo
*Outcomes*	In depth interviews A (n = 10) and informal discussions with Pasteur Institute, local mosquito control, health & Project staff used to develop questionnaire for household survey, and content of presentations, stakeholder contact list and future interviews with residents.			
***Baseline data – quantitative.***	Household survey (n = 100) residents. Aims: demographic information (name, age, gender, occupation, education level); identify important local health issues; lay knowledge of dengue, its vectors, control methods, and disease risk; and early responses to the *Wolbachia* strategy	Local residents TNI	Random sample.	Analysis in SPSS
*Outputs*	*Draw on results from activities above to:* Develop community profile and stakeholder contact list; Develop presentation for future focus group style meetings with Residents and Leaders; Develop in depth interview guide for future interviews with Residents; Modify communication materials			
***Phase 3: feedback and update Leaders finalize CP, SL and comm. Materials.***	2^nd^ Workshop with Leaders Feedback on progress and science. Sought further advice/input on nature and form of engagement, authorization, communication materials, & responses to *Wolbachia* strategy	Local & district leaders, government representatives, health providers & mosquito. control staff.	By invitation. Purposive, those in leadership roles in govt. and health	Responses recorded. Anon. questionnaire distributed and analyzed - SPSS.
*Outputs*	Finalize TNI community profile and stakeholder contact list. Finalize presentation for future meetings/workshops with Residents and Leaders. Finalize in-depth interview guide for future interviews with Residents Finalize communication materials			
***Phase 3. Research with residents.*** Aims: Inform, identify questions and concerns, acceptability, engagement and authorization	January 2010, Community meetings TNI residents.	Members of local unions, leaders and health staff	Sampled purposively by invitation.	Audio recorded Anon. questionnaire
	March 2010, Community meetings TNI Residents and Residents interviews (n = 10).	Local Residents and Leaders TNI	Random sample: participants are invited using flyers and through announcements over the loud speaker in the community.	Audio recorded Field notes NVivo Anon. quest. SPSS
	May 2010, Community meetings TNI and NT Residents and Residents interviews (n = 10)	Local Residents & Leaders TNI & NT		
	July 2010, Community meetings, TNI Residents	Local Residents and Leaders TNI		
*Outputs*	Draw on results from above activities to: Identify and include any new insights, questions, concerns or calls for more information into presentations, flyers and communication materials. See results section for details.			
***Phase 4. Update on progress of social research, science and feedback results.***	3rd Workshop with Leaders. Updated on progress and results of both social and scientific research. Sought further advice/input on regulatory approval process and responses to *Wolbachia* strategy	Same as LFG #2	Purposive. Invitation.	Responses recorded. Anon. quest. distributed and analyzed
*Outputs*	Draw on results from above activities to: Identify and include any new insights, questions, concerns or calls for more information into presentations, flyers and communication materials. See results section for details.			
***Phase 5. Design engagement strategy***	Draw on results from all activities to: Create a formal engagement strategy: which includes key stakeholders identified in the research and engages at least one householder from each home on TNI; Finalize communication materials, flyers, presentations for future use; Continue to liaise with govt officials, feedback community responses. See results section for details. A*wait outcomes of VN Govt. regulatory approvals for a release.*			

### Research: Process and methods

In the following section we describe the methods used at each step in the research process and how the key results were used to design an engagement framework and communication materials tailored to this potential release site. We do so on the assumption that successful engagement leading to a release using new dengue control methods is still somewhat rare and that it is the process as much as the results that will be of interest to others looking to engage communities around new disease control strategies.

The first step in the process was to immerse the two social science staff in the science of dengue and the *Wolbachia* strategy and to identify any information about the history and demographics of the potential release site. This included an extensive literature review on dengue fever, bio-control, GM food and organisms in Vietnam and internationally, and the development of a database (Table 1).

In June 2009 a PowerPoint presentation was developed (Table 2). It used the same slides and followed the same narrative structure as the presentation used at the Australian field site, to which Vietnam specific information was then added. Graphics with small amounts of text were used to communicate key messages around the following themes: increasing prevalence of dengue (local, national, international); disease transmission and vectors; current control measures in Vietnam; the *Wolbachia* strategy; the Australian pilot release; a potential release on TNI. A discussion was then facilitated to identify any questions or concerns and seek guidance on how to engage, whom to engage, what would constitute authorization (Table 2).

**Table 2:** Key themes of presentations to residents and leaders.

Themes	Slides
Dengue prevalence	Increase in disease incidence over time, internationally and in VN; Dengue fever in VN and TNI - most cases and deaths are in the south
Transmission cycle and local vectors	What is dengue fever? ; How do you get dengue fever?; Symptoms and signs; Vectors, habits and habitats
Current control measures	Review current control measures –Vietnam. “There is currently no known vaccine or cure for dengue fever”; “The challenge for scientists is to develop new strategies to prevent the mosquito from transmitting dengue fever”
The *Wolbachia* project	The Research team, Scientific collaborators in VN, Funding bodies. Project Aim: to Eliminate Dengue by more natural means.
A New approach: the *Wolbachia* strategy	What is *Wolbachia*?; What are its known effects?; The presence of bacterium in many local insects. The Idea: introduce *Wolbachia* to *Aedes aegypti* mosquito, describe effects: viral interference; life shortening; egg viability; bendy proboscis. Highlight implications of these for dengue transmission.
Australian pilot release	Introduced bacterium to the mosquito – effects; Caged trials: purpose of; Independent Risk Assessment – Australia – results; Approval for a release: Australian Government and local Communities
A future pilot release on TNI?	Explain: our desire to consult with the community, to seek their input and guidance about a possible future release on TNI. Why TNI has been identified as ideal for a pilot release. What are the caged trials and population studies in Vietnam for? What a pilot release would entail: suppression, release, population replacement, monitoring; Importance of authorization from the community and Government. *We want to hear any questions, thoughts or concerns. We want to learn how we should engage, who and when we should engage, and what would constitute authorization.*
Facilitate Discussion	In later presentations new results from social and scientific research in Vietnam were added to the presentation.

In July 2009 this presentation was used at the first of three leaders workshops, with thirty national, provincial, district and commune leaders, scientists and local health providers in attendance. Participants were chosen purposefully, because of their roles as leaders or officials and formally invited to attend. They included Ministry of Health leaders and scientists, members of the Khanh Hoa People's Committee and Khanh Hoa Health Department, and community and union leaders from TNI and NT. Project scientists and social scientists from Vietnam and Australia were present at the workshop.

At the first Leaders Workshop (Hanoi, July 2009) project employees were introduced to participants, and presentations delivered on the impact of dengue fever in Vietnam, the science behind the *Wolbachia* method, the potential release strategy in Vietnam and progress at the Australian release site (scientific and engagement). The presentation was approximately 20 minutes long, after which a discussion was facilitated while the second social scientist made observations on body language; interactions between participants and audio recorded the entire event - presentation and discussion. Participants were asked if they had any questions, thoughts or concerns and what their expectations around the strategy, engagement and authorization might be. Input was also sought to identify key stakeholders as well as feedback on the presentation and project communication materials.

An anonymous questionnaire was distributed at the end of the leaders workshop. It asked participants to identify any concerns or questions, evaluate how acceptable the *Wolbachia* strategy was, how they wished to be engaged and what would constitute authorization. This questionnaire provided baseline data for evaluating responses to the *Wolbachia* strategy through time, and was an important mechanism for tracking responses to the project among the leaders group and later, local residents. This process was also used at the Australian field site [6].

In early September 2009, a senior entomologist working for the *Wolbachia* project, who was well known to the local community, introduced project staff to Tri Nguyen (TNI) residents. Limited information on the history and demographics of TNI was publically available so a purposive sample of 10 in-depth interviews on the history, socio-political structure, social demographics and dengue history of TNI was undertaken with local residents and leaders. Purposive sampling involves the deliberate selection of individuals because of the crucial information they can provide – in this case local leaders with a detailed knowledge of the history and socio-political make up of the TNI community. These interviews, alongside informal discussions with local health and mosquito control staff and results from the Leaders workshop, were used to develop a detailed stakeholder contact list, which was added to over time. It categorized individuals and groups according to: level of influence (local, national, international); local expectations around engagement; marginality; and accessibility. This helped to determine who was engaged and when. In addition, results from the interviews were also used to improve the PowerPoint presentation and communication materials to be used at future community meetings and workshops.

In the next stage of the process, the results from these interviews were used to develop a Household Survey that examined the following: political structure (leaders, groups, organizations); social demographics of TNI (name, age, gender, occupation, education level, religion, family structure); knowledge of dengue, its vectors, control methods and perceptions of risk; and local health issues of concern to residents. The survey provided a brief introduction to the *Wolbachia* strategy and sought to identify early responses and advice on engagement and authorization for a release. The survey was piloted with 10 residents, reviewed and later administered to 100 households randomly selected from a list of 710 provided by local authorities - approximately 14% of all households.

The second Leaders Workshop was held in the mainland city of Nha Trang, and attended by 33 participants representing local (TNI) and district leaders, government representatives, scientists, local health providers and mosquito control staff. An update on the progress of the science, the Australian risk assessment and the release was provided and further advice sought on stakeholders, forms of engagement, authorization and the presentation and communication materials. As noted above, a discussion was facilitated and any questions or concerns were noted. The event was also audio-recorded for later transcription and analysis and the anonymous questionnaire distributed.

During the next phase of the project, 46 community meetings, attended by 661 local residents, were held in TNI during four, one-week trips in January (T1), March (T2), May (T3) and July (T4) 2010 (Table 1). The aim of these meetings was to gauge the range of views on the *Wolbachia* strategy, the science, potential release, engagement and authorization using the same focus group style format as the Leadership Workshops. Discussion was facilitated around the following themes: questions raised, concerns, acceptability, how and whom to engage and authorization. The meeting was audio-recorded and the anonymous questionnaire distributed at the end (Table 1).

During the second visit (March 2010) local residents who had contracted dengue attended the meeting and spoke of their experiences during the presentation. In addition, new results from the independent Australian Risk Assessment and new experiments showing *Wolbachia* was not transmitted to predators who ingested the infected mosquitoes were added to the presentation. During the third (May 2010) and fourth visit (July 2010), results of Vietnamese experiments indicating that ingesting infected mosquitoes did not affect or lead to transmission of *Wolbachia* among local predatory species was included. By this time we also had more information about government approval processes (following the final Leaders Workshop) and the likely time frame for this, so this to was incorporated into presentation. Other than these additions, the presentation was the same at each visit.

For the community meetings on TNI, a small number of participants were approached directly and sampled purposefully (i.e. health staff, hamlet and local union leaders and members) based on the stakeholder list we had begun developing. However, the majority of participants were sourced through flyers, posters and announcements over the community loudspeaker prior to each visit. As such the sample was broadly representative, with participants self-selecting to be involved. We aimed to reach at least one person from every TNI household (Table 1).

During the second (March 2010) and third (May 2010) visits, 20 in-depth interviews were also undertaken with residents from TNI and NT (aged 18–60 years) who could not attend the meetings. We approached marginalized or harder-to-reach groups identified during the Leaders Workshops and early interviews (n = 10) with local leaders. This included fishermen who were often away from the island, women with domestic and employment duties and minority religious or ethnic groups who it was thought might otherwise not have been engaged. These interviews began with the PowerPoint presentation and explored the same issues as the workshops and meetings. They were audio recorded for transcription purposes.

The third and final Leaders Group Meeting was held in Nha Trang and attended by 33 local, district and national leaders, local health and mosquito control staff and scientists. Presentations on the results of both the social and scientific research were provided, and further advice sought on regulatory pathways and approval processes in Vietnam. The anonymous questionnaire was also distributed.

Two social scientists and at least one senior entomologist attended every meeting or workshop. Prior to any research or engagement, an extensive and detailed list of questions and answers posed by the public at the Australian field site, was made available to Vietnamese project staff. It was posted to the project's website in June 2009 (see http://www.eliminatedengue.com/faqs for the current version) and later, on the Vietnamese language version and developed into flyers provided to participants. As the research progressed, it was clear that this extensive list covered almost every question posed by participants in the Vietnam research. When new questions or issues did arise, they were answered, if possible. If it was not possible to answer a question, it was recorded so that a response could be sought from appropriate staff and later provided back to the person asking the question and the community. This practice helped to ensure that information across the field sites, project staff and research activities - meetings, workshops, interviews etc. - was accurate and consistent.

## Results

Results from the in-depth interviews (n = 10) and Household survey identified three active civic groups on TNI: the Women's, Youth and Farmer (includes fishing) Unions. They were well respected in the community and would in all likelihood, be central to future research activities as well as an important conduit for disseminating information. They were given priority in the engagement framework that was being developed. The Household survey indicated that 29% of those surveyed identified as ‘Buddhist’, and 71% as ‘Non-religious’. In addition, 89% of adults surveyed (over 18 years) had a primary or secondary education, 6% had completed high school and 5% self identified as non-literate. Fishing (a predominantly male occupation) was the primary source of income for 70% of households surveyed, with small scale trading enterprises providing income for 13%. Women ran most of these. Average monthly incomes per household ranged from up to i) 2,000,00 DN (USD $95) 39%; ii) from 2,001,000 (USD $96) to 4,000,000 (USD $190) 37%; iii) from 4,001,000 (USD $191) to 6,000,000 (USD $285) 16% and iv) more than 6,000,000 DN (USD $285) at 6%. In sum, 76% of households surveyed earned up to 4,000,000 DN (USD $191) per month, based on exchange rates in September 2009.

Participants from the leaders interviews (N = 10) and workshops advised that a presentation delivered in a meeting and styled on a focus group - like the one they had attended - was in fact an appropriate way to communicate the *Wolbachia* story to TNI residents. They also recommended project staff work through the highly structured networks of governance identified above and undertake extensive consultations with local health staff, unions and residents at TNI. In the Household Survey, TNI residents concurred with this finding, suggesting project staff work through local hamlet and union leaders who in turn would inform residents. As one local resident expressed it: “When we want to know any information, the first persons we always come to are the local leaders such as: hamlet leaders, women union's leader…. I think that they are in charge of responding to any issues related to our local community” (interview, TNI resident, 42-year-old woman). Many residents explained that the role of the hamlet and union leaders was not only to represent them, but also to inform and connect local people with community activities – an important finding for developing an engagement framework.

Interviews with local hamlet leaders and health workers signposted that they expected to be engaged early and regularly, so meetings with these individuals and groups were given priority in the research phase and the final engagement strategy.

We [local health staff] also expect to be updated on the project in order to contribute to or be involve in any required situation such as any problems or urgent crisis issues happening during the implementation of this project” (interview, male staff member, 35 years old, Vinh Nguyen Health Station).

Residents also expected to be widely consulted about the strategy:

…you should provide further explanation about the method, the procedure to apply the method …. You should ask as many people to share their opinion as possible; local authorities should also be consulted (interview, male, 49 years old, TNI resident)

In addition, results from the Household survey, interviews (n = 10) and leaders workshops provided a number of insights critical to understanding the multiple ‘publics’ at the potential release site. They were used to prepare a comprehensive community profile, and develop a stakeholder contact list, the later categorized and prioritized groups according to local expectations (i.e. health workers and union officials to be engaged early), marginality (i.e. women and religious groups) and accessibility (i.e. fishermen). It began to emerge that an engagement strategy for TNI would need work through established political structures and engage at least one person from every household.

### Examining lay knowledge of dengue, its vector/s, its management and biological control

Results from the Household survey (n = 100) indicated that residents were well versed on prevention activities and current control methods, i.e. covering water containers, insecticide use, bed nets etc. [20]. Although 65% of those surveyed correctly identified key domestic breeding sites, there was also a strong and recurring association between ‘dirty places’, namely sewers, forested areas, and refuse and the mosquitoes thought to transmit dengue. Although 65% were able to identify the mosquito primarily responsible for dengue transmission in TNI, only 35% were able to explain the transmission cycle or describe symptoms – both of which were central to understanding the *Wolbachia* strategy (for more details see Huong and McNaughton 2012.

The Household Survey (n = 100) revealed that most residents (93%) identified dengue fever as a dangerous disease within their community. The main reasons cited were that it can be fatal (83.9%) and can spread very fast (40.9%). Residents looked first to local health workers (95%), followed by television (55%) and local officials (41%) as trusted sources of information on dengue and health. These and other results were used to develop a more targeted PowerPoint presentation on the *Wolbachia* strategy that focused on symptoms, the transmission cycle and the habitats of the vectors, three key gaps in local understandings. This presentation was used at 46 focus-group style meetings with 661 residents (Table 1).

### Residents meetings: Safety, efficacy, responsibility and impact

The most prominent and recurring issue for respondents across the residents' and leaders' meetings and interviews was the safety of the method for people, animals and the environment. Relatedly, participants wanted to know if it was safe to be ‘bitten’ by a *Wolbachia*-infected *Ae. aegypti* mosquito, if was safe to drink water with these mosquitoes, their larvae or pupae in it, and if this would lead to *Wolbachia* being transmitted into other organisms, especially people. For example, a member of the youth union asked “Is it a problem if we are bitten by *Wolbachia*-infected mosquitoes? Can *Wolbachia* be transmitted into our body?” Some also expressed concerns that *Wolbachia*-infected mosquitoes might become susceptible to or able to transmit other diseases: “After releasing the *Wolbachia*-carrying mosquitoes, the dengue fever may be reduced, but how about other diseases; will it cause any other disease to come to our Island?”

Responses to questions relating to the potential transmission of *Wolbachia* to humans, other organisms or the environment included but were not limited to the following:

Yes, it is safe to be bitten by mosquitoes carrying *Wolbachia pipientis*. *Wolbachia* cannot be transmitted to humans or any vertebrates. The transmission of *Wolbachia* between insect species is thought to occur very rarely in nature. It lives inside the insects (hosts) cells and tissues, it cannot survive outside of them. This makes it almost impossible for it to be transmitted to other insect species, including those that might harbor disease. Also, it is naturally occurring with up to 70% of all insect species, including in many mosquitoes that bite people, and insects that humans have eaten for a long time.

A discussion about the role of many project staff in blood feeding large numbers of these mosquitoes in the caged trials and laboratories (including photos) often ensued.

Alongside safety, considerable discussion centered on why TNI had been chosen as a potential release site, if it would be the first to trial this strategy and who would be responsible if anything should go wrong. For example, “I heard many people who participated in your discussions ask each other why this method was not applied somewhere else but on Tri Nguyen Island. Is it safe if it is applied here?” (Male, 25 years, member of the Youth union). Another resident expressed concerns about safety and responsibility as follows:

There are not many DF cases on the island, only 3–5 cases a year. If mosquitoes are released … and cause some problems such as raising the number of DF cases to 50 or 70, will the project be responsible for the problem? Will the project have any commitment with local people? Will they have some commitment to ensure there are no problems? (Community Meeting CM, T1).

For many participants, assurances were sought that Australia rather than Vietnam would be the first place to release these mosquitoes. In addition, residents wanted clear pathways of responsibility outlined so they knew whom to speak to should something go wrong. Several residents asked directly, “Which agency will be in responsibility in case the release strategy will cause additional impacts?” (CM, T3). Local government and health officials also wished to know who would be responsible in the event of any problems and sought greater clarity from each other and project staff and leaders, regarding their specific responsibilities during a pilot release.

Clear lines of responsibility had been established and these were relayed to residents with responses like the following:

Professor Nguyen Tran Hien, Director of the National Institute of Health and Epidemiology is responsible for monitoring the project's activities and managing the responsibilities of project partners. Local health partners such as Khanh Hoa health department, Nha Trang health center have also been invited to monitor any health related issues during the release and after. We will also set up a hotline and an office on TNI, so local people can come and discuss or report any concerns or questions they have.

Another common concern centered on the efficacy of the strategy, especially in the long term. One resident attending the group asked “…does it [*Wolbachia*] have any side effects after being introduced into mosquitoes? It is a bacterium, so it must be harmful to some extent”. (CM, T2). Many participants were also concerned that the life shortening effect of *Wolbachia* would impact on the success of the strategy, “How can *Wolbachia*-infected mosquitoes help prevent the disease when they die early after being released?” (CM, T1). “I am concerned that it may be difficult for *Wolbachia*-infected mosquitoes to find another mosquito to copulate with, or that they may die before they can lay their eggs” (CM, T2). Many participants were interested in eliminating all mosquitoes or why current control methods were no longer as viable: “Why don't you try to kill all mosquitoes? Why don't you spray chemicals to kill them all?”(CM, T3). The 2009 Household Survey (n = 100) had indicated that while 86% found the *Wolbachia* strategy acceptable, the use of insecticides either inside (67%) or outside (74%) their homes was also viewed positively (see Table 3).

**Table 3:** Acceptability of mosquito control methods, Household Survey 2009.

Which methods would you find acceptable?	Acceptable	Unacceptable	Undecided
Spraying insecticide inside your home	67	31	2
Spraying insecticide outside around your home	74	24	2
Releasing *Wolbachia*-infected mosquitoes	86	1	13
Introducing *Mesocyclops* to mosquito breeding containers	70	6	24
Releasing genetically modified mosquitoes that cannot transmit dengue to people	64	7	28

Responses to questions relating to efficacy, focused in part on the role of the trials in determining the effectiveness of this strategy, and that results from the Australian releases would be reported back to the community during future engagement. They also included, but were not limited to, the following (for more details http://www.eliminatedengue.com/faqs):

Scientists hope that by introducing this life-shortening strain of *Wolbachia* bacterium into *Ae. aegypti*, the mosquitoes will die before they are old enough to transmit dengue virus to people. There will still be *Ae. aegypti* mosquitoes in the environment, they will live and breed as normal but they won't live as long. Reducing the number of old mosquitoes will disrupt the transmission cycle of dengue. Another effect of the *Wolbachia* bacteria is to distort the reproductive success of the mosquitoes in favour of those with the *Wolbachia*. This means that the *Wolbachia* will spread more rapidly throughout the mosquito population. Also, *Aedes aegypti* mosquitoes mate 2–3 days after they emerge and can blood feed by day 2–4. After 6–8 days they can lay eggs, so the younger *Aedes aegypti* mosquitoes produce most of the young. So removing the oldest mosquitoes from the population should not significantly reduce the overall size of the mosquito population and research in the field cages at James Cook University in Australia show that *Wolbachia* mosquitoes can through mating, eventually replace wild mosquito populations. In a field trial, reducing the wild population before a release will give them the best chance. We will be providing more details about the results of the Australian release in the future. If we were to release here on TNI we would be monitoring the situation, trapping mosquitoes and seeing how many have *Wolbachia* and we would be updating the community about this.

The nature and scale of the pilot release were also prominent, recurring issues from the community meetings and interviews (n = 20). Respondents commonly sought a high level of detail regarding the release, its timing and scale. Questions focused on further details regarding how many mosquitoes would be released, if this would be in all or only some houses, and how long it would take for wild mosquitoes to be infected. There was a lot of discussion about what residents should do to assist the effectiveness of the strategy and what impact this might have on people's lives. For example:

In the early stage of the release, both *Aedes aegypti* and *Wolbachia*-infected mosquitoes live in our environment. What we should do to avoid being infected with DF? Will the project support us if we have DF then?” (CM, woman age 28, member of Youth union)

When you release *Wolbachia*-infected mosquitoes to the island, how can we distinguish them from normal mosquitoes? Is it a problem if we kill them?” (CM, male, aged 57, member Farmers union).

This question was answered as follows:

We cannot distinguish them from normal mosquitoes; to our eyes they look the same. It is OK if local people kill the *Wolbachia* mosquitoes in nets or with rackets; we encourage local people to keep their behaviors and practices as normal. At the time of the release and while we are monitoring it, we would ask that residents not use insecticide, but that is only a request.

During the Residents meetings (n = 46) and interviews (n = 20) assurances were often sought that the release would not negatively affect or inhibit local lives and livelihoods and that householders would be made aware of any activities they needed to undertake before or during a release. There was strong support for being advised and informed well in advance of a release “so that we are well prepared for it?” (CM, T2).

In general, we responded to these questions as follows:

We want to ensure that we do not disturb the lives of local householders and we encourage all local people to keep their habits and practices. Before the release, the project will send a newsletter to all households to let them know when the release is occurring and how we will monitor it. We also have a team of collaborators who will visit local households every week we release and to help communicate all necessary information.

**Figure 2:**
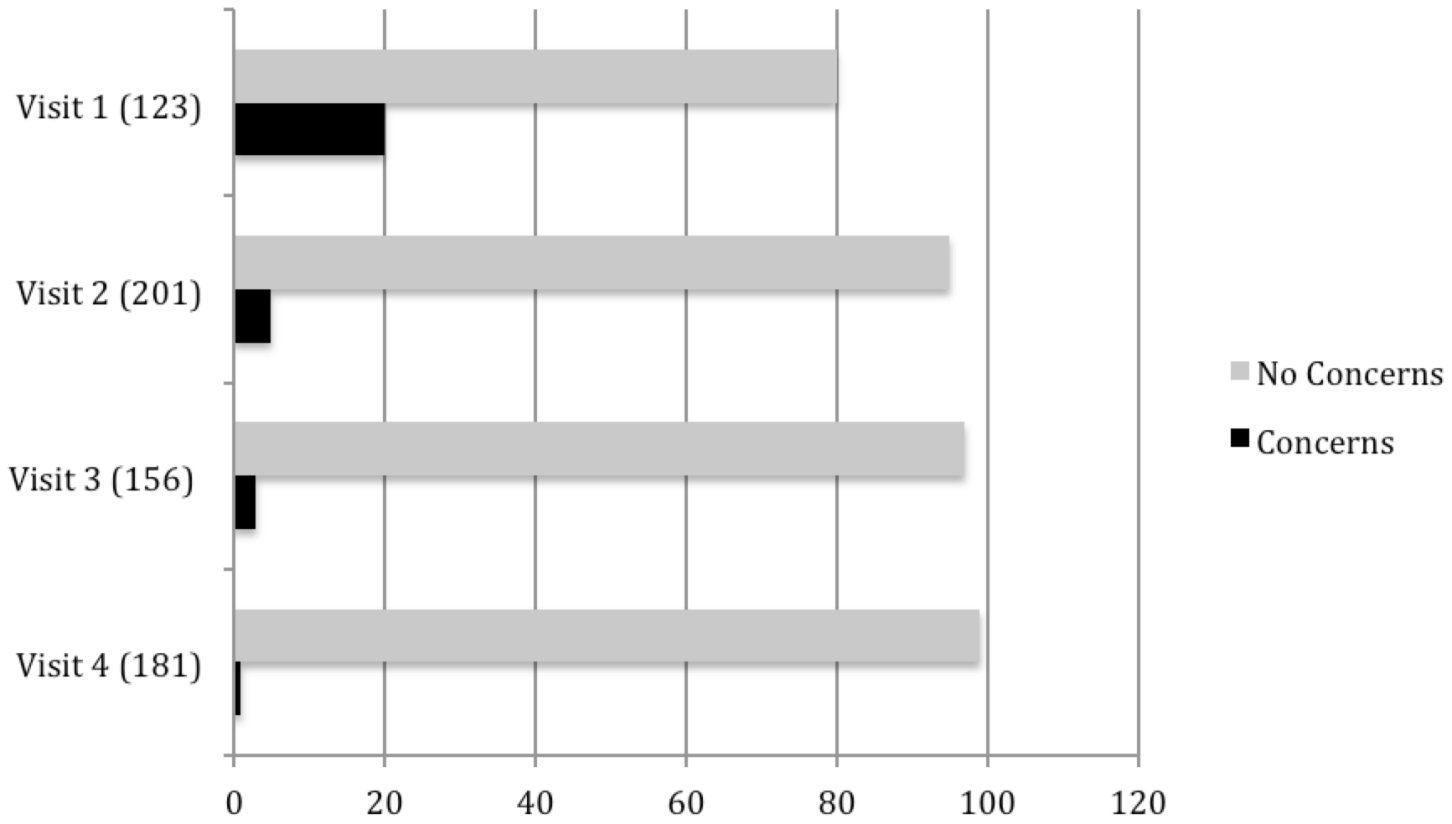
Participant concerns about the *Wolbachia* method. Participant responses to the question ‘Do you have any concerns about the *Wolbachia* method?’ (Sample size shown in brackets).

The anonymous questionnaire, handed out at the end of each meeting included the question, “Do you have any concerns about the *Wolbachia* method?” which was used to track residents' perceptions of the project through time. As indicated in Figure 2, the number of concerned participants declined significantly as the Residents' Meetings and interviews continued. During the final two visits to TNI in May and July 2010, no participants objected to a release (Figure 2).

### How the multiple publics want to be engaged and what would constitute authorization for a release

Participants were asked at the Leaders workshops (n = 3), Residents' Meetings (n = 46) and interviews (n = 20) how they would like to be engaged about the *Wolbachia* strategy. There was a strong desire for public consultation across all groups, consistent support for in-community presentations and a strong preference for face-to-face interaction with the project team and senior health officials. There was much less support for the use of media, posters, brochures and leaflets.

One of the most common requests related to the scale of the engagement. At the local level, participants consistently indicated that well before a release the project team should engage with *every* community member and provide ongoing information on the safety and benefits of the project well before a release. For example, “More people, all people should be invited. A small group of participants like this is not representative enough to make a decision. It is perfect if 100% of people agree” (CM, T3). Others suggested that, at minimum, one person from each household should be engaged. For example, “One person from every household should be invited. The main income earner in every household should be invited so that they can remember what they have heard and tell others. If you invite those who are too old, they may not have a good memory to tell others about what they have heard” (CM, T3).

Participants were also asked what would be the best format to engage people on TNI about the strategy and in the lead up to a release if regulatory approval was given. There was an expectation of ongoing consultation about the strategy among residents, leaders and health staff, where updates on the science, safety, risk assessment, regulatory approval, pilot release strategy, results from the Australian release and a well-defined structure around roles and responsibilities would be provided. Some were also concerned that without this, people might forget what they had learned about the strategy and how to respond to a release. Community leaders and health professionals suggested that residents would come to them for information and guidance, especially if things did not go to plan. As such they sought to have clear pathways on any future roles and responsibilities they might have negotiated, outlined and communicated to residents well before a release.

As well as calling for regular updates, participants consistently identified the importance of a large meeting attended by at least one representative from each household as well as local and provincial leaders – essentially a forum where people could raise their ideas, discuss benefits and concerns and make a collective decision (Table 4). There was also a strong preference for voting at such a forum, as one resident expressed it “Voting can be used. Those who agree will raise their hand. If the majority raises hands that means it is supported” (CM, T1) (Table 5). As such a large public meeting held in the community or a vote was identified as a mechanism through which the project and the release would gain final and collective approval from the TNI community, alongside support of government officials (regulators, Ministry of Health and scientists) (Table 5).

**Table 4:** Mechanisms for informing and engaging residents at TNI, Household Survey 2009.

Best ways to inform and engage residents at TNI	Yes	%	No	%	Total
Give presentation to community groups	201	70%	86	30%	287 (100%)
Informing and working with local leaders	184	64%	103	36%	287 (100%)
Informing and working with health workers	177	62%	110	38%	287 (100%)
School education program	65	23%	222	77%	287 (100%)
Public meetings	213	74%	74	26%	287 (100%)

**Table 5:** Mechanisms for gaining public authorization at TNI, Household Survey 2009.

Mechanisms for gaining public authorization	Yes	%	No	%	Total
Public meetings	169	64%	94	36%	263 (100%)
Approval by local leaders	133	51%	130	49%	263 (100%)
Approval by Vietnamese government	158	60%	105	40%	263 (100%)
A vote on Tri Nguyen Island	169	64%	94	36%	263 (100%)

**Figure 3:**
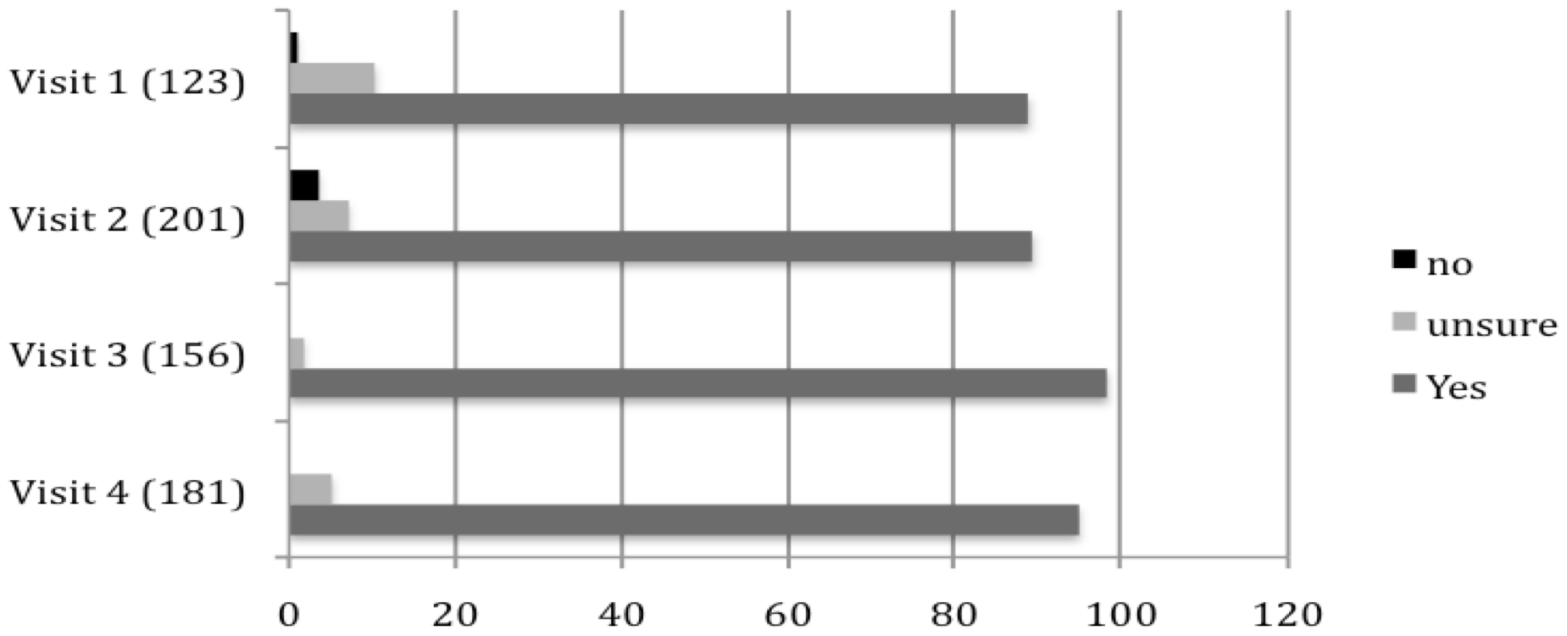
Participant support for release of *Wolbachia* infected *Aedes aegypti* mosquitoes. Participant responses to the question ‘Would you support the release of *Wolbachia* infected *Aedes aegypti* mosquitoes as a means of controlling dengue fever in your community if (a) the Ministry of Health undertook a risk assessment and approval process, and (b) the scientific data from the Australian release site proved to be positive?’ (Sample size shown in brackets).

The anonymous questionnaire also asked whether Resident's would support a pilot release if (a) the Ministry of Health undertook a risk assessment and approval process, and (b) scientific data from the Australian release site proved to be positive. During the first phase of social research and engagement in January 2010, 80.2% were in favor of the pilot release. By the final phase in July 2010, this had risen to 99.4% (Figure 3). Of course, participants can and do change their minds and they could react differently when a release happens, and this is a limitation of this study.

However, results from the Australian research did allow us to predict quite successfully how people would react and there was no last minute call to stop the release in Australia. Although a release has not yet occurred in Vietnam, the most recent engagement with TNI residents (2013) - where one person from every household was interviewed - 99% of householders were still in favor of the release, only a few months shy of its eventuality (data not shown).

## Discussion

Participants in this study brought different forms and degrees of knowledge about science, dengue fever, its vectors and control to their encounter with the *Wolbachia* strategy. While breeding sites and control measures were relatively well known, and many participants connected mosquitoes to dengue fever, few people were able to explain the dengue transmission cycle. Understanding the transmission cycle, and the role of *Wolbachia* in blocking the development of the dengue virus, was essential to understanding the strategy, perceived risks and ensuring residents were well informed. Addressing these gaps and assumptions was given high priority in presentations and communication materials used in research phase and in the development of a TNI engagement framework. It was essential to ensure as much as possible, that the project communicated the *Wolbachia* story as accurately, effectively and transparently as possible so that community members were able to engage, critique and ultimately decide whether they wish to support such an initiative. As the quotations provided above indicate, a good understanding of the strategy was evident among many respondents.

The research identified a range of concerns regarding the safety of *Wolbachia* for people, animals and the environment and in particular, the potential for transmission of the bacterium through biting behavior or accidental ingestion (the later unique to the Vietnamese field site). Identifying these concerns well in advance of a release (or a formal engagement strategy) provided an opportunity to develop clear, consistent responses to these issues that were comprehensible to local populations.

Although the scientific team was confident about the safety of the strategy, new experiments examining the potential for *Wolbachia* to be passed into the human bloodstream through the mosquito's saliva during feeding [5] as well as testing *Wolbachia's* capacity to be transferred from mosquitos to predator and non-predator species such as spiders, fish, copepods and geckos, common to local environments were undertaken [21]. Findings from these studies were incorporated into the community presentation and communication materials and fed back to residents during the research phase. Results of an independent Australian risk assessment, which suggested the risk to people and the environment were negligible, were also added. This, coupled with confirmation that the *Wolbachia* strategy would be trialed first in Australia, demonstrated to residents that their concerns about safety and the location of the first field trial had been taken seriously. This was important to the success of the approach and to future engagement.

Results from the social research suggested that procedures for consulting communities were well established on TNI and this involved consulting first with leaders at the national, provincial, and hamlet levels before moving out into the community. Indeed, it appeared that a similar process was expected for any research or future engagement seeking community support and authorization. Most people we spoke to wanted the community to come together as a group or, as representatives of individual households, determine the benefits and risks and decide through a vote or similar mechanism, whether or not to support a release. It emerged that although the role of leaders, government officials and scientists in decision making was important to many residents, so too was the role of local residents in deciding household by household, on whether or not to use this strategy. This we learned, was the process most residents thought should be used to seek their support and authorization for a release. This was the approach that was taken the formal engagement phase post 2010.

The growing acceptability of the *Wolbachia* strategy and a release over the research phase suggests that this approach was effective (Figure 3). Engagement from 2011–2012 drew on all of the findings and lessons highlighted above. Following an update on the latest results from the science and the first field trials in Australia [22] a representative from *every* household on TNI was asked to provide their consent, or not, for a release. Of these, more than 95% agreed to support the release. In 2013 the Vietnamese government gave regulatory approval for an open field release in TNI.

### Conclusion

The approach described here produced a number of critical insights that helped determine the nature, scale, style and form of an engagement framework tailored specifically to the needs and wishes of officials and residents and the potential release site in Vietnam. It used systematic social research and consultation to (a) identify, inform and involve the public; (b) listen to their responses, questions and concerns; (c) examine the deeper cultural assumptions that underwrite these responses, including lay knowledge of dengue; (d) explore ways of responding to these issues i.e. scientifically, through education, the media, schools programs or new forms of participation; and (e) explore and enact suggestions regarding future engagement, participation, communication and authorization.

Through this process we found that residents at the potential release site in Vietnam expected to be fully informed and fully engaged about the science, the project, its safety, risk assessments, the nature of the release and who would be responsible should something go wrong. Along with key health and government officials and representatives they provided advice on how best to engage their community and wanted the opportunity to meet with and ask questions of scientists involved in these programs and to have their concerns taken seriously and answered respectfully. This approach thus afforded the development of a more culturally appropriate and comprehensible engagement framework and communication materials that empowered those being asked to assess, critique and support a field trial or release. It has now been implemented at three socially and politically diverse and complex field sites (seven in Australia, one in Vietnam) in two countries, demonstrating its capacity to reflect local requirements and its potential for use in other programs and other regions.
